# Surgical outcomes of Glaucoma associated with Axenfeld-Rieger syndrome

**DOI:** 10.1186/s12886-020-01417-w

**Published:** 2020-05-01

**Authors:** Emily M. Zepeda, Kari Branham, Sayoko E. Moroi, Brenda L. Bohnsack

**Affiliations:** Department of Ophthalmology and Visual Sciences, Kellogg Eye Center, University of Michigan, 1000 Wall Street, Ann Arbor, MI 48105 USA

**Keywords:** Axenfeld-Rieger syndrome, Iridogoniodysgenesis, Pediatric glaucoma, Secondary glaucoma, Angle surgery

## Abstract

**Background:**

The surgical management of glaucoma associated with Axenfeld-Rieger Syndrome (ARS) is poorly described in the literature. The goal of this study is to compare the effectiveness of various glaucoma surgeries on intraocular pressure (IOP) management in ARS.

**Methods:**

Retrospective cohort study at a university hospital-based practice of patients diagnosed with ARS between 1973 and 2018. Exclusion criterion was follow-up less than 1 year. The number of eyes with glaucoma (IOP ≥ 21 mmHg with corneal edema, Haabs striae, optic nerve cupping or buphthalmos) requiring surgery was determined. The success and survival rates of goniotomy, trabeculotomy±trabeculectomy (no antifibrotics), cycloablation, trabeculectomy with anti-fibrotics, and glaucoma drainage device placement were assessed. Success was defined as IOP of 5-20 mmHg and no additional IOP-lowering surgery or visually devastating complications. Kaplan-Meier survival curves and the Wilcoxon test were used for statistical analysis.

**Results:**

In 32 patients identified with ARS (median age at presentation 6.9 years, 0–58.7 years; median follow-up 5.4 years, 1.1–43.7 years), 23 (71.9%) patients were diagnosed with glaucoma at median age 6.3 years (0–57.9 years). In glaucomatous eyes (46 eyes), mean IOP at presentation was 21.8 ± 9.3 mmHg (median 20 mmHg, 4-45 mmHg) on 1.0 ± 1.6 glaucoma medications. Thirty-one eyes of 18 patients required glaucoma surgery with 2.2 ± 1.2 IOP-lowering surgeries per eye. Goniotomy (6 eyes) showed 43% success with 4.3 ± 3.9 years of IOP control. Trabeculotomy±trabeculectomy (6 eyes) had 17% success rate with 14.8 ± 12.7 years of IOP control. Trabeculectomy with anti-fibrotics (14 eyes) showed 57% success with 16.5 ± 13.5 years of IOP control. Ahmed© (FP7 or FP8) valve placement (8 eyes) had 25% success rate with 1.7 ± 1.9 years of IOP control. Baerveldt© (250 or 350) device placement (8 eyes) showed 70% success with 1.9 ± 2.3 years of IOP control. Cycloablation (4 eyes) had 33% success rate with 2.7 ± 3.5 years of IOP control. At final follow-up, mean IOP (12.6 ± 3.8 mmHg, median 11.8 mmHg, 7-19 mmHg) in glaucomatous eyes was significantly decreased (*p* < 0.0001), but there was no difference in number of glaucoma medications (1.6 ± 1.5, *p* = 0.1).

**Conclusions:**

In our series, greater than 70% of patients with ARS have secondary glaucoma that often requires multiple surgeries. Trabeculectomy with anti-fibrotics and Baerveldt glaucoma drainage devices showed the greatest success in obtaining IOP control.

## Background

Axenfeld-Rieger Syndrome (ARS), a rare congenital disease that affects approximately 1 in 200,000 live births, is characterized by eye and craniofacial defects [1–4]. Within the eye there is a specific set of anterior segment anomalies: 1) Anterior displacement of Schwalbe’s line (posterior embryotoxon), 2) Adherent iris strands to the posterior embryotoxon (Axenfeld Anomaly), and 3) Iris hypoplasia that results in corectopia and/or pseudopolycoria (Rieger Anomaly) [5–8]. While these entities can be isolated findings or individually associated with other eye conditions, the combination of them constitute ARS [1, 9].

The anterior segment dysgenesis and craniofacial abnormalities (telecanthus, maxillary hypoplasia, microdontia, and oligodontia) in ARS are commonly a result of genetic disruption of neural crest cell migration and differentiation due to autosomal dominant mutations within the *PITX2* or *FOXC1* genes [4, 10–15]. Various studies have demonstrated that mutations in either gene are associated with early-onset glaucoma [16, 17].

Glaucoma is reported to complicate approximately 50% of ARS cases and is the main cause of vision loss [1, 16] The elevated intraocular pressure (IOP) in ARS is due to inherent iridogoniodysgenesis and iris strands that bridge the angle structures [1, 3]. This secondary glaucoma is often refractory to medications requiring surgery to obtain IOP control. However, due to the rarity of ARS, there are few reports in the literature regarding the surgical management of this form of glaucoma [1, 18–23]. Further, there is disagreement amongst the few reports as to the effectiveness of angle surgery in ARS [3, 21–23]. In the current study, we review our 45-year experience with ARS-associated glaucoma and specifically focus on the success of surgical management in order to obtain IOP control.

## Methods

A retrospective chart review identified patients with a diagnosis of ARS that presented to the Kellogg Eye Center between January 1, 1973 and January 1, 2018. Exclusion criterion was follow-up of less than 1 year. This study was approved as exempt by the Institutional Review Board at the University of Michigan.

Collected data included age, gender, ocular and systemic findings, intraocular surgeries, dates of surgeries, surgical procedure details, and intraoperative and postoperative complications. Visual acuities, IOPs, and ocular medications were recorded for each visit. Preoperative IOP and ocular medications were last recorded values before surgery. Visual acuity was determined using age and developmentally appropriate measures. In non-verbal individuals, vision was assessed by ability to fixate on and follow objects and ocular preference. Visual acuity as measured by optotypes was converted to LogMAR scale. A greater than 2-line difference in visual acuity was considered significant when comparing initial and final values. IOPs were measured by Icare® (Revenio, Vantaa, Finland), Tono-pen® (Reichert, Depew, NY, USA) or Goldman applanation.

The diagnosis of ARS was made clinically based on the criteria listed in the background. Glaucoma was defined by at least 2 repeated IOP measurements greater than 21 mmHg and accompanying signs of corneal edema, Haabs striae, optic nerve cupping, or buphthalmos. Success of glaucoma surgery was defined as IOP of 5–20 mmHg and no additional IOP-lowering surgery or visually devastating complications.

Glaucoma surgical procedures, including goniotomy, trabeculotomy, combined trabeculotomy and trabeculectomy, contact transcleral cyclophotocoagulation, endoscopic cyclophotocoagulation, trabeculectomy with anti-fibrotics, and glaucoma drainage device placement, were generally performed using standard techniques [24–30]. Briefly, goniotomy was performed with a 25-gauge needle or Barkan knife [31]. Trabeculotomy was performed from a superior or temporal approach with Harms trabeculotomes. For combined trabeculotomy and trabeculectomy, the trabeculotomy was completed with Harms trabeculotomes and then aqueous humor egression from under the scleral flap was adjusted using 10–0 nylon sutures [22]. For contact transcleral cyclophotocoagulation, the ciliary body was treated 180 to 270 degrees with the G-probe (Iridex, Mountain View, CA, USA) and diode 810 nM laser (Iridex). Endoscopic cyclophotocoagulation was performed with the E3 Endo Optiks unit (Little Silver, NJ, USA), targeting 180 to 270 degrees of ciliary body. For trabeculectomies with anti-fibrotics, 50 mg/ml 5-fluorouracil or 0.2 to 0.4 mg/ml mitomycin C was applied to the scleral bed through pledglets for 2 to 5 min or subconjunctivally injected prior to incision. Flow through the trabeculectomy flap was adjusted in the first 1–2 postoperative weeks using laser suture lysis. Implantation of glaucoma drainage devices was performed as previously described [24]. The choice of Ahmed© FP7 or FP8 (New World Medical, Rancho Cucamonga, CA, USA), or Baerveldt© 101–250 or 101–350 (Abbott Medical Optics, Santa Ana, CA, USA), was based on surgeon preference, preoperative IOP, extent of glaucoma damage, and eye size. For Baerveldt© 101–350 or 101–250 (Abbott Medical Optics) devices, the tube was either temporarily ligated with a 6–0 polyglactin suture or implanted in 2 stages separated by at least 4 weeks [32, 33].

GraphPad Prism 7.0 (GraphPad, La Jolla, CA, USA) was used for statistical analyses including Kaplan Meier survival curves with 95% confidence interval (CI) and Wilcoxon test for initial and final comparisons. The reported *p*-values were two-tailed. *P* values less than 0.05 were considered statistically significant.

## Results

Thirty-two patients with a diagnosis of ARS (41% male) presented at a median age of 6.9 years (mean age 14.4 ± 16.3 years, range 0.0–58.7 years) and were followed for a median 5.4 years (mean 9.2 ± 10.9 years, range 1.1–43.7 years). Nine patients in 5 families underwent genetic testing and were found to have *FOXC1* mutations (Supplemental Fig. 1). Three individuals within 1 family had a *PITX2* mutation, which was previously reported by our group [34]. In addition to the family with the *PITX2* mutation, fourteen patients had a family history of ARS of which 11 patients comprised 5 different families while 3 patients had affected family members not included in this study (Supplemental Fig. 1).

Twenty-three patients were diagnosed with bilateral glaucoma at a median age of 6.3 years (mean age 11.6 ± 15.0 years, range 0.0–57.9) years. There were no patients who had unilateral glaucoma. Thirty-one eyes (67% of eyes with glaucoma) of 18 patients required glaucoma surgery with a mean of 2.2 ± 1.2 (median 2, range 1–6) surgeries per eye. In eyes with glaucoma, the mean IOP at presentation (21.8 ± 9.3 mmHg, median 20 mmHg, range 4–45 mmHg), was significantly higher than the mean IOP at final follow up (12.6 ± 3.8 mmHg, median 11.8 mmHg, range 7–19 mmHg), (*p* < 0.0001). The mean number of glaucoma medications was not significantly different at presentation (1.0 ± 1.6, median 0, range 0–6) compared to final follow-up (1.6 ± 1.5, median 1.5, *p* = 0.1).

**Fig. 1:**
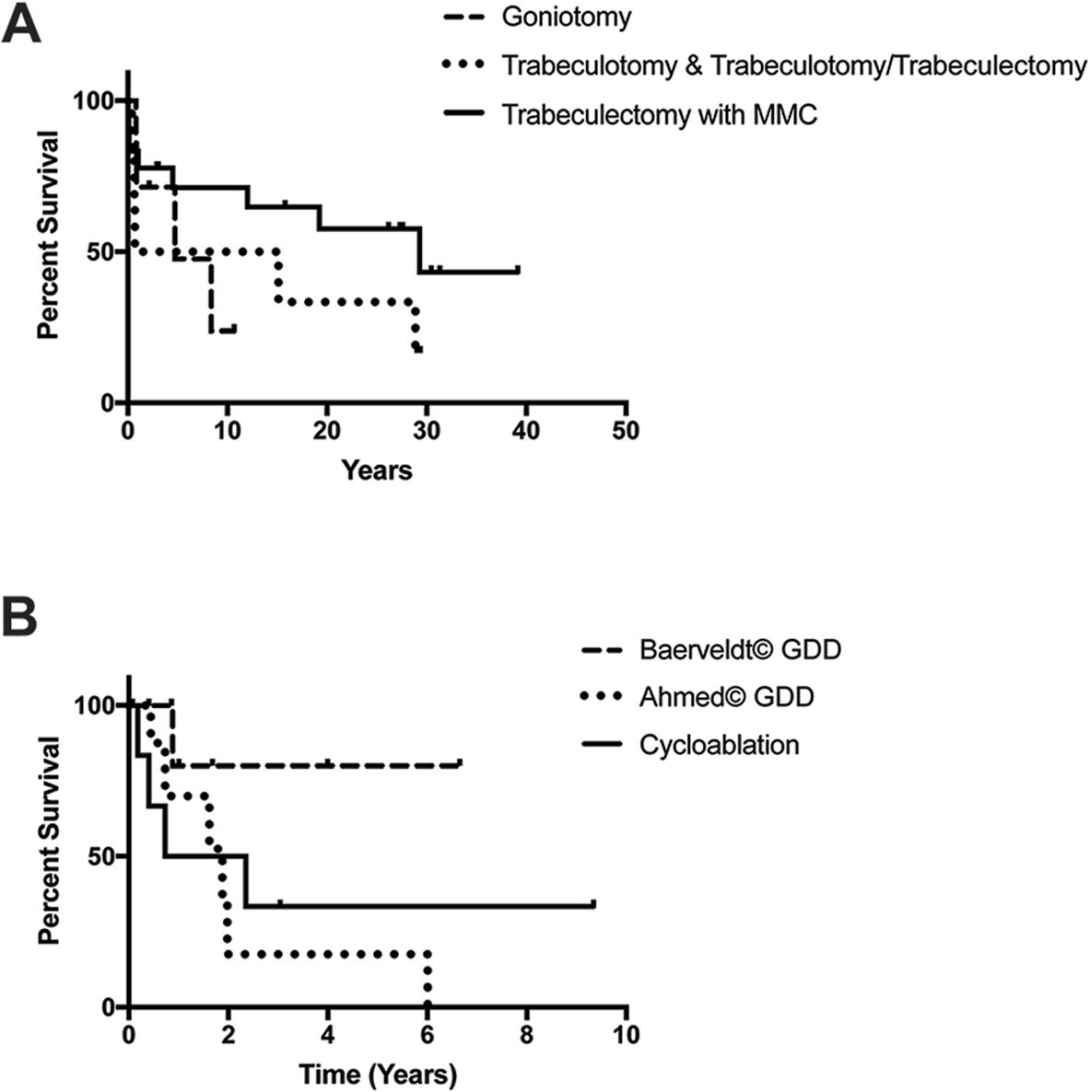
Glaucoma Surgery Outcomes. **a** Kaplan Meier analysis demonstrated goniotomy had survival rates of 71% with 95% CI [26, 92] at one year, 48% with 95% CI [8, 81] at five years, and 24% with 95% CI [1, 64] at 10 years. Trabeculotomy or combined trabeculotomy and trabeculectomy showed survival rates at 1 year of 50% with 95% CI [11, 80], 15 years of 33% with CI [4, 67] and 25 years of 17% with 95% CI [1, 52]. Survival rates of trabeculectomy with anti-fibrotics (C) were 78% with 95% CI [51, 91] at one year, 65% with 95% CI [38, 83] at 10 years and 43% with 95% CI [14, 69] at 30 years. **b** Ahmed© FP7 or FP8 GDDs had a survival rate of 70% with 95% CI [23, 92] at one year and 18% with 95% CI [1, 53] at 2 years. Baerveldt© 101–250 or 350 GDDs showed a survival rate at one year of 75% with 95% CI [33, 93] and five year of 61% with 95% CI [21, 86]. Contact or endoscopic cycloablation had survival rates of 50% with 95% CI [11, 80] at one year and 33% with 95% CI [4, 67] at five years

The glaucoma surgeries performed in the 31 eyes with ARS are summarized in Table 1. Goniotomy had survival rates (Fig. 1a) of 71% with 95% CI [26, 92] at one year, 48% with 95% CI [8, 81] at five years, and 24% with 95% CI [1, 64] at 10 years. Trabeculotomy or combined trabeculotomy and trabeculectomy showed survival rates (Fig. 1a) at one year of 50% with 95% CI [11, 80], at 15 years of 33% with CI [4, 67] and at 25 years of 17% with 95% CI [1, 52]. At final follow-up, goniotomy was more successful (50%) than trabeculotomy or combined trabeculotomy and trabeculectomy (17%). The mean years of IOP control following goniotomy (4.3 ± 3.9 years, median 2.2 years, range 0.9–10.7 years) was not statistically significant different (*p* = 0.06) than trabeculotomy or combined trabeculotomy/trabeculectomy (14.8 ± 12.7 years, median 15.1, range 0.4–28.9 years).

**Table 1:** Surgical Management of ARS and Outcomes

Glaucoma Surgery • Total number of type of glaucoma surgery • Number of eyes of number of patients • Average age at time of surgery in years	Previous Glaucoma Surgery (Number of Eyes)	Next Additional Glaucoma Surgery (Number of Eyes)	Years of IOP Control (Range)	Success at Final Follow-up
Goniotomy • 7 Total Goniotomies • 6 Eyes of 3 Patients • 1.7 ± 1.4 years	4 Eyes of 2 Patients • No Prior Surgeries 3 Eyes of 2 Patients • Prior Goniotomy (1) • Prior Trabeculotomy/Trabeculectomy (2)	3 Eyes of 2 Patients • Trabeculectomy w/anti-fibrotics (3)	4.3 ± 3.9 (2.2-10.1)	• 43% for goniotomy • 50% for eyes due to multiple goniotomies in 1 eye
Trabeculotomy or Combined Trabeculotomy/ Trabeculectomy • 6 Total Trabeculotomies ± Trabeculectomies • 6 Eyes of 4 Patients • 3.3 ± 7.4 years	6 Eyes of 4 Patients • No Prior Surgeries	5 Eyes of 3 Patients • Trabeculectomy w/anti-fibrotics (3) • Baerveldt© 350 (1) • Goniotomy (1)	14.8 ± 12.7 (0.4-29.2)	• 17% for trabeculotomy ± trabeculectomy
Trabeculectomy with Anti-Fibrotics • 18 Total Trabeculectomies with Anti-Fibrotics • 14 Eyes of 8 Patients • 16.1 ± 14.8 years	8 Eyes of 5 Patients • No Prior Surgeries 10 Eyes of 7 Patients • Prior Trabeculectomy (4) • Prior Trabeculotomy (2) • Prior Goniotomy (2) • Prior Trabeculotomy and Goniotomy (1) • Prior Ahmed, Baerveldt© 250, Cycloablation (1)	6 Eyes of 5 Patients • Repeat Trabeculectomy (3) • Bleb Revision (2) • Baerveldt© 350 (1)	16.5 ± 13.5 (0.1-39.1)	• 57% for trabeculectomy • 71% for eyes due to multiple trabeculectomies in 3 eyes
Ahmed© Glaucoma Drainage Device FP7 (6) and FP8 (3) • 9 Total Ahmeds© • 8 Eyes of 5 Patients • 7.2 ± 7.0 years	8 Eyes of 5 Patients • No Prior Surgery 1 Eye of 1 Patient • Prior Ahmed (1)	6 Eyes of 4 Patients • Cycloablation (1) • Baerveldt© 250 (3) • Baerveldt© 350 (1) • Tube Revision (1)	1.7 ± 1.9 (0.4-6.0)	• 25% for Ahmed© GDDs
Baerveldt© Glaucoma Drainage Device 250 (3) 350 (8) • 11 Total Baerveldts© • 9 Eyes of 6 Patients • 16.8 ± 11.7 years	2 Eyes of 1 Patient • No Prior Surgery 7 Eyes of 5 Patients • Prior Trabeculotomy (1) • Prior Cycloablation (1) • Prior Ahmed© (4) • Prior Ahmed©, Cycloablation (1) • Prior Ahmed©, Baerveldt© 250 (2)^a^	3 Eyes of 4 Patients • Endoscopic cycloablation (1) • Baerveldt© 350 (2)^a^	1.6 ± 2.1 (0.2-6.7)	• 70% for Baerveldt© GDD • 88% for eyes due to multiple Baerveldt© GDDs in 2 eyes
Cycloablation • 6 Total Cycloablations • 4 Eyes of 3 Patients^b, c^ • 16.0 ± 7.8 years	4 Eyes of 3 Patients • Prior Ahmed© (3) • Prior Baerveldt© 350 (1)	3 Eyes of 2 Patients^b, c^ • Repeat Cycloablation (2) • Baerveldt© 350 (2)	2.7 ± 3.5 (0.4-9.4)	• 33% for cycloablation • 50% for eyes due to multiple cycloablations in 2 eyes

^a^2 eyes with inferonasal Baerveldt© 250 GDDs had subsequent removal of superotemporal Ahmed© FP7 devices and placement of superotemporal Baerveldt© 350 GDDs

^b^1 eye had endoscopic cycloablation followed by contact transcleral cycloablation

^c^1 eye had 2 sessions of contact transcleral cycloablation followed by Baerveldt© 350 placement

Survival rates of trabeculectomy with anti-fibrotics (Fig. 1a) were 78% with 95% CI [51, 91] at one year, 65% with 95% CI [38, 83] at 10-years, and 43% with 95% CI [14, 69] at 30 years. Further, trabeculectomy with anti-fibrotics showed a 57% success rate with a mean of 16.5 ± 13.5 years (median 17.5 years, range 0.1–29.3 years) of IOP control. However, 3 eyes had repeat trabeculectomies leading to a 71% success rate for the eyes that underwent filtering surgeries. One eye that underwent trabeculectomy with mitomycin C developed bleb-related endophthalmitis.

Ahmed© FP7 or FP8 glaucoma drainage devices had a survival rate (Fig. 1b) of 70% with 95% CI [23, 92] at one year and 18% with 95% CI [1, 53] at two years. The success rate at final follow-up of the 8 eyes that had Ahmed© glaucoma drainage device placement was 25% with a mean of 1.7 ± 1.9 years (median 1.2 years, range 0.4–6.0 years) of IOP control. Baerveldt© 101–250 or 350 GDDs showed a survival rate (Fig. 1b) at one year of 75% with 95% CI [33, 93] and 5-year of 61% with 95% CI [21, 86]. At final follow-up, Baerveldt© glaucoma devices had a 70% success rate with a mean of 1.6 ± 2.1 years (median 1.0 years, 0.2–6.7 years) of IOP control, but 2 eyes had multiple Baerveldt© glaucoma drainage devices resulting in an 88% success rate in eyes that underwent Baerveldt© implantation.

Cycloablation (transcleral or endoscopic) was performed in 4 eyes with an average of 1.5 ± 0. 6 treatment per eye (range 1–2). All 4 eyes had undergone prior glaucoma surgery (3 had prior Ahmed© glaucoma drainage device placement and 1 had prior Baerveldt© 350 glaucoma drainage device placement). Survival rates of cycloablation (Fig. 1b) were 50% with 95% CI [11, 80] at one year and 33% with 95% CI [4, 67] at five years. Cycloablation had a 33% success rate with a mean of 2.7 ± 3.5 years (median 1.5, range 0.4–9.3 years) of IOP control, but 2 eyes had multiple cycloablations resulting in a 50% success rate in eyes that underwent cycloablation.

**Fig. 2:**
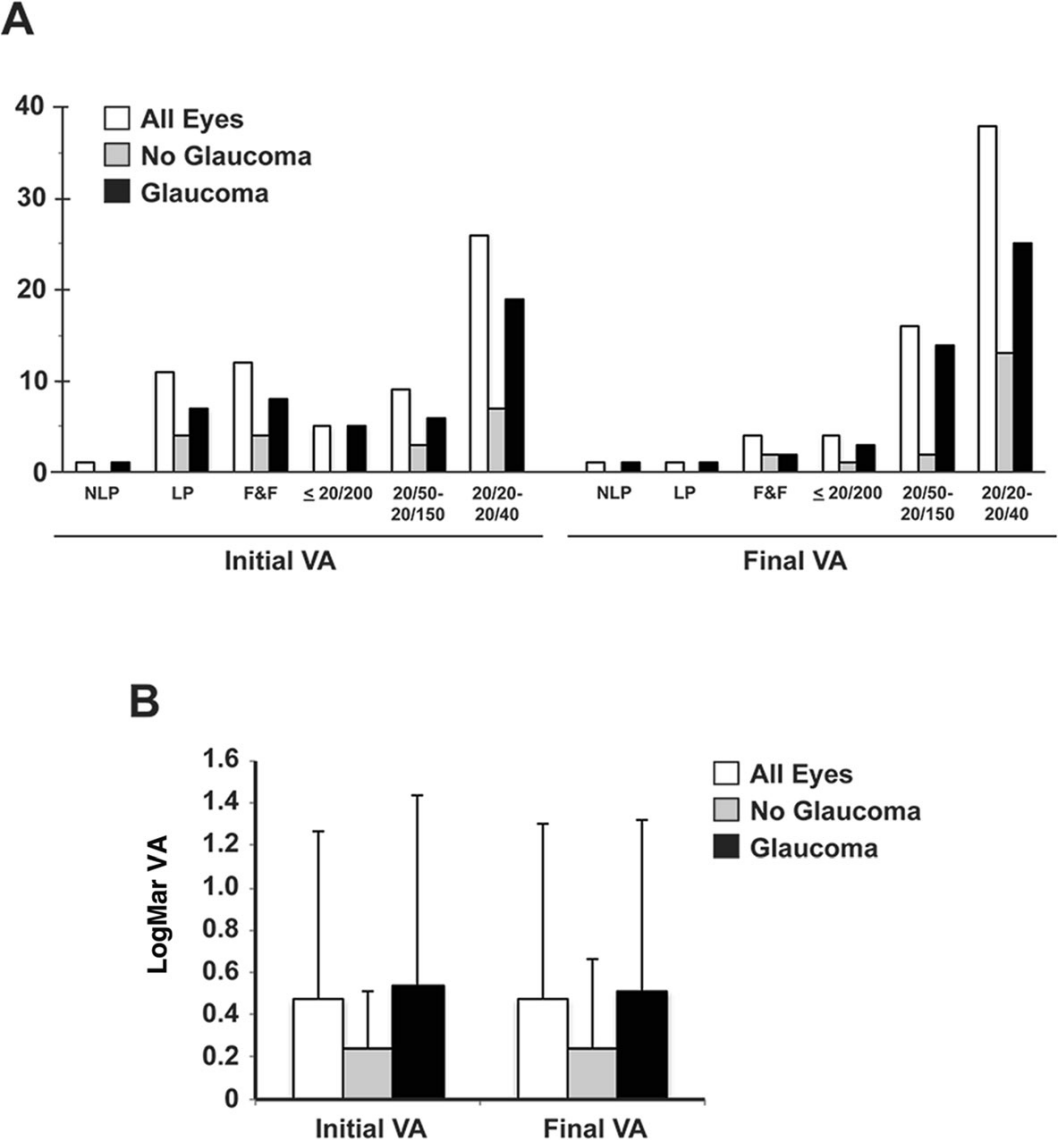
Visual Acuity Outcomes. **a** A greater number of eyes, with and without glaucoma, showed light perception or fix and follow vision due to age at initial presentation. At final follow-up, 54 (79%) eyes had visual acuity better than 20/200 with 38 (59%) with visual acuity of 20/40 or better. **b** In eyes in which optotype testing was obtained, the average LogMAR visual acuity at presentation (0.5 ± 0.8) and final follow-up (0.5 ± 0.8) was not different (*p* = 0.81). In eyes with or without glaucoma, LogMAR visual acuity at presentation (0.5 ± 0.9, 0.2 ± 0.4, respectively) and final follow-up (0.5 ± 0.8, 0.3 ± 0.4, respectively) was not significantly different (*p* = 0.85, *p* = 0.99, respectively). The visual acuity in eyes without glaucoma was not significantly different compared to eyes with glaucoma at presentation (*p* = 0.30) and at final follow-up (*p* = 0.21)

In this study, measurement of visual acuity was limited by age in 12 patients at presentation and in 2 patients at final follow-up. Despite this limitation, 60 of the 64 eyes had stable or improved vision at final follow-up (Fig. 2a). The 4 eyes of 3 patients that had worse vision all required glaucoma surgery, and 3 of the eyes underwent either partial (Descemet stripping automated endothelial keratoplasty, 2 eyes) or full thickness (penetrating keratoplasty, 1 eye) corneal transplantation. In eyes in which optotype testing was obtained, the mean LogMAR visual acuity at presentation (0.5 ± 0.8, median 0.2) and final follow-up (0.5 ± 0.8, median 0.2) was not statistically different (*p* = 0.81) (Fig. 2b). In eyes with glaucoma, mean LogMAR visual acuity at presentation (0.5 ± 0.9, median 0.3) and final follow-up (and 0.5 ± 0.8), median 0.3 were not significantly different (*p* = 0.85). Further, mean LogMAR visual acuity in eyes without glaucoma was not different at presentation (0.3 ± 0.4, median 0.1) compared to final follow-up (0.2 ± 0.4, median 0.1, *p* = 0.99). There was no statistically significant difference in mean LogMAR visual acuity between glaucomatous eyes and non-glaucomatous eyes at presentation (*p* = 0.30) or at final follow-up (*p* = 0.21).

## Discussion

ARS is characterized by a specific set of anterior segment anomalies that is associated with glaucoma in approximately half of affected individuals [1–4, 6, 9, 16]. Despite the prevalence of glaucoma, the most effective surgeries to decrease IOP in ARS are debated within the literature [1, 18–23]. In the current study, we reviewed our experience with the surgical management of glaucoma in patients with ARS over a 45-year period. We found that 72% (23/32) of patients with ARS had glaucoma, all of which were bilateral. This higher percentage of glaucoma in our study may be attributed to the referral pattern and tertiary level of care at our institution. However, the true rate of glaucoma may be difficult to ascertain as ARS findings may be subtle and go undiagnosed. For example, 2 of our patients were only diagnosed when their children were found to have glaucoma secondary to ARS. Thus, family members of affected individuals should be carefully screened for ARS.

Although it is generally accepted that this form of glaucoma is often refractory to medications, the percentage of eyes with ARS that require IOP-lowering surgery is not well-defined. Previously published studies either concentrate on the genetics of the individuals with ARS or focus on the eyes that required glaucoma surgery [1, 4, 10–15, 18–23]. In our study, we found that 67% of eyes with glaucoma and 48% of all eyes with ARS required IOP-lowering surgery. While this is a high percentage, one-third of eyes with glaucoma did not required IOP-lowering surgery at final follow-up. Thus, unlike primary congenital glaucoma, medications should still be the first-line treatment to lower IOP in ARS.

There are few reports on the surgical management of glaucoma in ARS, but in general, most eyes with ARS require multiple surgeries in order to achieve IOP control [1, 18–23]. Our findings were consistent with this as eyes with glaucoma had on average 2.2 ± 1.2 glaucoma surgeries. The need for multiple surgeries may reflect the refractory nature of this glaucoma as well as the lack of agreement on best surgical procedures for lowering IOP. The greatest debate revolves around the effectiveness of angle surgery in ARS. Histopathologic studies from Shields suggested that angle surgery would not be successful in ARS [3]. In previous reports, goniotomy has a success rate ranging from 0 to 40%, and has the additional disadvantage of requiring a gonioscopic view [21, 23]. In our study, goniotomy had a 43% success rate at final follow-up, but only a 24% survival rate at 10 years. However, goniotomy showed higher success and survival rates than trabeculotomy alone or combined with trabeculectomy (33% survival at 15 years and 17% success rate at final follow-up). This may be due to the longer time of IOP control in eyes that had undergone trabeculotomy ± trabeculectomy (mean 14.8 years) compared to goniotomy (mean 4.3 years) as well as surgeon experience with the different angle surgeries. While other studies have also shown that trabeculotomy has poor success in ARS [1, 18, 19], trabeculotomy combined with trabeculectomy (without anti-fibrotics) in an Indian population was recently found to be effective in obtaining IOP control with an 88% survival at 5 years [22]. This contrast in results may be due to differences in surgical procedure or in inherent population variations. Although angle surgery is the mainstay for many pediatric glaucomas, our results suggest that goniotomy and trabeculotomy are not be sufficiently effective in obtaining long-term IOP control in ARS.

Trabeculectomy with anti-fibrotics has been previously shown to have the greatest success in glaucoma associated with ARS [1, 18, 19]. However, some of these reports are over 30 years old, prior to the introduction of glaucoma drainage devices. Similar to these studies, we found that trabeculectomy augmented with either mitomycin C or 5-fluorouracil has a 57% success rate at final follow-up with 8 blebs lasting greater than 20 years. Nonetheless, the life-long risk of bleb-related infections, especially in children, must be considered when performing this procedure [35–38]. In our study, 1 of the 14 eyes, which underwent trabeculectomy with anti-fibrotics, had a bleb-related endophthalmitis. This child presented with glaucoma at birth and underwent trabeculectomy with mitomycin C that effectively decreased IOP. At 5 years of age, the child had endophthalmitis resulting in permanent loss of vision from 20/40 to worse than 20/400. Although his vision at final follow-up measured better than his initial vision (light perception), the bleb-related infection took its toll on his vision. Thus, as with other forms of glaucoma, trabeculectomy with anti-fibrotics can obtain long-term IOP control, but may not be the best option for young children due to the lifelong risk of visually-devastating infections.

Few studies with only a scant number of eyes have assessed the effectiveness of glaucoma drainage devices in ARS [19]. In our study, we found that Baerveldt© 101–350 glaucoma drainage devices were successful in obtaining IOP control compared to Baerveldt© 101–250 and Ahmed© glaucoma drainage devices. In our hands, Ahmed© devices showed poor success rate (25%), despite the fact that none of the 8 implanted eyes had a history of prior glaucoma surgery. In 6 of the 8 eyes, the blebs encapsulated between 0.4 to 6 years, necessitating additional surgery, which was most often implantation of a Baerveldt© device. The 2 Ahmed© glaucoma drainage devices that remained successful at follow-up were implanted with Ologen© (Aeon Astron, Leiden, Netherlands) biodegradable collagen matrix which is theorized to prevent bleb encapsulation [39]. The Baerveldt© 101–250 glaucoma drainage devices were all placed inferonasally as a follow-up surgery from superotemporal Ahmed© placement. The inferonasal placement, beneath the medial rectus, inferior rectus, and inferior oblique muscles, may limit the surface area of the bleb and decrease the IOP-lowering effect. The 7 eyes that had Baerveldt© 101–350 glaucoma drainage device placement showed an 86% success rate, however the length of follow-up is much shorter than trabeculotomy/traeculectomy and trabeculectomy with anti-fibrotics. Additional longitudinal studies are required to fully assess the effectiveness of glaucoma drainage devices in ARS.

In the current study, cycloablation (transcleral or endoscopic) was not used as a primary glaucoma surgical intervention, but utilized in eyes in which a glaucoma drainage device (3 Ahmed© and 1 Baerveldt©) had failed. Following cycloablation, 2 of the 4 eyes underwent replacement of the Ahmed© for a Baerveldt© 350 glaucoma drainage device. Although the success rate for cycloablation itself was 33%, the combination of glaucoma drainage devices and cycloablation (in some cases multiple sessions) was successful in obtaining IOP control in all 4 eyes. Thus, cycloablation is useful in refractory cases of glaucoma secondary to ARS.

With adequate IOP control, previous studies have shown that individuals with ARS typically have visual acuity better than 20/50 [22]. In our study, we found that the average LogMAR visual acuity at final follow-up was 0.47 (between 20/50 and 20/60). Although ARS eyes with glaucoma trended toward worse visual acuity at presentation and final follow-up compared to eyes without glaucoma, the difference was not statistically significant. However, it is important to note that visual acuity measurements were limited by age, especially at presentation. Nevertheless, we found that 95% of all eyes (61 of 65 eyes) maintained or showed improvement in vision at final follow-up compared to presentation. The 4 eyes that had at least a 2-line loss of visual acuity at final follow-up all had glaucoma and required IOP-lowering surgery (2.5 ± 1.2 surgeries/eye). In addition, 3 of the 4 eyes underwent either partial or full-thickness corneal transplantation for endothelial dysfunction or corneal opacification. Thus, eyes with ARS overall had good visual outcomes regardless of glaucoma diagnosis.

The strengths of our study are the relatively high number of eyes and patients given the rarity of ARS and the 45-year length of the study. Limitations include the retrospective nature of this case series, variable time of follow-up, lack of control population, and variable disease severity at initial presentation. In addition, due to the length of the study, these procedures were performed by 5 surgeons who had different criteria for surgical intervention and various levels of expertise in the surgical procedures. Further, given the changes in glaucoma surgery over the past 4 decades, the surgeons also had different surgical procedures at their disposal resulting in longer follow-up for angle surgeries and trabeculectomies with anti-fibrotics compared to glaucoma drainage devices. Although the study included 32 patients, 18 of whom required glaucoma surgery on one or both eyes, the total number of eyes in each glaucoma surgical category is small. A prospective trial with a higher volume of patients is difficult due to the rarity of the disease, although a multi-center collaborative study could help to address this problem.

In conclusion, our case series demonstrates that glaucoma associated with ARS often requires multiple surgeries. We found that angle surgery in our hands is less effective in obtaining long-term IOP control. Filtering surgery with anti-fibrotics shows good success with the longest follow-up, but the lifelong risk of infection, especially within a pediatric population, must be considered. Baerveldt© 101–350 glaucoma drainage devices also obtain IOP control, however, additional follow-up is required to fully assess long-term success.

## Conclusions

In our series, greater than 70% of patients with ARS have secondary glaucoma that often requires multiple surgeries. Angle surgery is not sufficiently effective in obtaining long-term IOP control in ARS. Trabeculectomy with anti-fibrotics and Baerveldt glaucoma drainage devices showed the greatest success in obtaining IOP control. Additional follow-up is required to fully assess long-term success.

## Supplementary information

**Additional file 1: Supplemental Figure 1.** Pedigrees and Genetic Testing of Families with ARS. Fourteen patients represented 8 families with ARS. An additional 3 patients in 1 family was previously published [34]. Families 1–4 and family 8 had multiple members included in the study. Individuals in families 5, 6 and 7 had other members with ARS not included in the study (demarcated by an asterisk) due to either deceased status or geographic location. Genetic testing was performed in all affected individuals in family 2 [*FOXC1* c.1491C > G (premature termination p.Tyr497*)], family 3 [*FOXC1* pHis128Pro:c.383A > C], and family 4 [1.72 Mb Deletion including *FOXC1* and few surrounding genes]. Genetic testing performed in 2 sporadic ARS cases identified *FOXC1* c1297_1298delCT and *FOXC1* c.821dupC, p.Ser276Glnfs*30 mutations.

## Data Availability

All data generated or analyzed during the current study are available from the corresponding author on reasonable request.

## Abbreviations

ARS: Axenfeld-Rieger Syndrome
IOP: Intraocular pressure
CI: Confidence Interval
GDD: Glaucoma Drainage Device
