# Supplementary figures and images for: Surgical outcomes of Glaucoma associated with Axenfeld-Rieger syndrome
Source: BMC Ophthalmol. 2020 May 1;20:172. doi: 10.1186/s12886-020-01417-w (PMC7193416; doi:10.1186/s12886-020-01417-w)

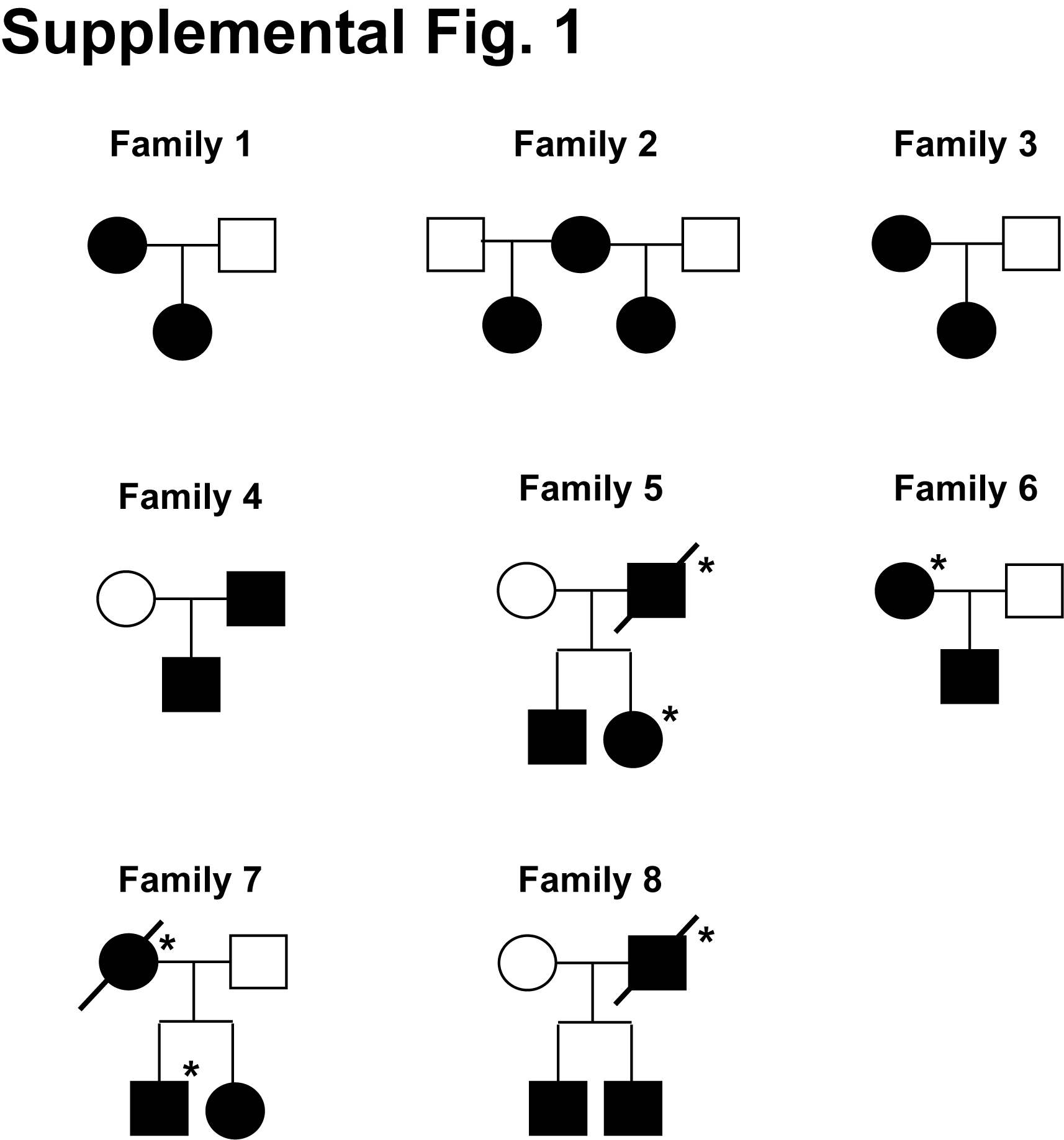

Supplement: Supplementary file 1 — Additional file 1: Supplemental Figure 1. Pedigrees and Genetic Testing of Families with ARS. Fourteen patients represented 8 families with ARS. An additional 3 patients in 1 family was previously published [34]. Families 1–4 and family 8 had multiple members included in the study. Individuals in families 5, 6 and 7 had other members with ARS not included in the study (demarcated by an asterisk) due to either deceased status or geographic location. Genetic testing was performed in all affected individuals in family 2 [FOXC1 c.1491C > G (premature termination p.Tyr497*)], family 3 [FOXC1 pHis128Pro:c.383A > C], and family 4 [1.72 Mb Deletion including FOXC1 and few surrounding genes]. Genetic testing performed in 2 sporadic ARS cases identified FOXC1 c1297_1298delCT and FOXC1 c.821dupC, p.Ser276Glnfs*30 mutations. [file 12886_2020_1417_MOESM1_ESM.tif]
